# Decellularized cartilage tissue bioink formulation for osteochondral graft development

**DOI:** 10.1088/1748-605X/ada59d

**Published:** 2025-01-13

**Authors:** Aleksandra A Golebiowska, Mingyang Tan, Anson WK Ma, Syam P Nukavarapu

**Affiliations:** 1 Department of Biomedical Engineering, University of Connecticut, Storrs, CT 06269, United States of America; 2 Department of Chemical and Biomolecular Engineering, University of Connecticut, Storrs, CT 06269, United States of America; 3 Polymer Program, Institute of Materials Science, University of Connecticut, Storrs, CT 06269, United States of America; 4 Department of Materials Science & Engineering, University of Connecticut, Storrs, CT 06269, United States of America; 5 Department of Orthopaedic Surgery, University of Connecticut Health, Farmington, CT 06032, United States of America

**Keywords:** decellularized cartilage, viscosity modifiers, bioactive biomaterial/ink, tissue engineering, articular cartilage, tissue bioink

## Abstract

Articular cartilage and osteochondral defect repair and regeneration presents significant challenges to the field of tissue engineering (TE). TE and regenerative medicine strategies utilizing natural and synthetic-based engineered scaffolds have shown potential for repair, however, they face limitations in replicating the intricate native microenvironment and structure to achieve optimal regenerative capacity and functional recovery. Herein, we report the development of a cartilage extracellular matrix (ECM) as a printable biomaterial for tissue regeneration. The biomaterial was prepared through decellularization and solubilization of articular cartilage. The effects of two different viscosity modifiers, xanthan gum and Laponite^®^, and the introduction of a secondary photo-crosslinkable component on the rheological behavior and stability were studied. dcECM-Laponite^®^ bioink formulations demonstrated storage modulus (G′) ranging from 750 to 4000 Pa, which is three orders of magnitude higher than that of the dcECM-XG bioink formulations. The rheological evaluation of the bioinks demonstrated the tunability of the bioinks in terms of their viscosity and degree of shear thinning, allowing the formulations to be readily extruded during 3D printing. Also, a spreadable ink composition was identified to form a uniform cartilage layer post-printing. The choice of viscosity modifier along with UV cross-linking warrants shape fidelity of the structure post-printing, as well as improvements in the storage and loss moduli. The modified ECM-based bioink also significantly improved the stability and allowed for prolonged and sustained release of loaded growth factors through the addition of Laponite^®^. The ECM-based bioink supported human bone-marrow derived stromal cell and chondrocyte viability and increased chondrogenic differentiation *in vitro*. By forming decellularized cartilage ECM biomaterials in a printable and stable bioink form, we develop a ‘Cartilage Ink’ that can support cartilaginous tissue formation by closely resembling the native cartilage ECM in structure and function.

## 1. Introduction

Osteochondral (OC) defect repair is a significant challenge and has attracted considerable interest due to the increase in osteoarthritis (OA). OA is a degenerative joint disease that causes progressive deterioration and irreparable damage to the OC tissue, including the articular cartilage and underlying subchondral bone. OA stands as the most common joint disease and cause of disability among adults in the US, affecting more than 60 million people and is responsible for over $300 billion annually in health care related costs [1]. The prevalence of OA is expected to continue to increase globally with the aging population, increasing rates of obesity and injury [2].

The common clinical treatment options for articular cartilage and OC defect repair include the use of autografts/allografts, microfracture and autologous chondrocyte implantation [3]. These techniques rely on accessing the mesenchymal stem cells (MSCs) that reside in the underlying bone marrow to participate in the regeneration process of the damaged cartilage. Another popular strategy is to harvest healthy autologous chondrocytes, and their expansion *in vitro* followed by re-implantation into the damaged site for repair. However, these treatment options have varied limitations such as donor-site morbidity, limited graft availability/insufficient integration, two-stage procedure, and dedifferentiation and phenotypic drift [4]. Moreover, the quality of the repaired tissue is also impacted and often lead to the formation of mechanically inferior fibrocartilage, rather than the functional hyaline cartilage, which breaks down and eventually may require multiple surgical interventions and total joint replacement [5-7].

Tissue engineering (TE) has emerged as a promising alternative strategy for the repair of damaged AC and OC tissues [8, 9]. TE strategies often focus on the development of implantable biomaterials alone or in combination with relevant cartilage/bone forming cells and/or growth factors [10]. Ideally, these biomaterial matrices should replicate the function of native extracellular matrix (ECM) and supply biochemical cues and signals to direct cellular behavior. Engineering a biomimetic system that provides a suitable microenvironment to recapitulate the complexity of the native ECM and contains necessary biological cues capable of directing cellular behavior holds great promise for improving tissue repair [11-14]. Moreover, due to the complexity of the OC tissue, where two distinct tissue types, articular cartilage and underlying subchondral bone, meet and form an interface, scaffold configuration is important to satisfy the requirements of both tissue types along with an interfacial zone. In particular, the use of multi-zonal scaffolds that takes into consideration of the individual phase requirements of the OC tissue with the formation of bi-phasic or tri-phasic scaffolds, and more recently gradient scaffolds that mimic the gradient transitions of the complex tissue (with gradual variation in biochemical composition, structure and physical properties along the graft length) has been gaining more attention [15-22]. Efforts in the literature have focused on developing OC grafts with the needed structural requirement, and grafts with growth factors and signaling molecules. There are no efforts where the OC graft biochemical composition and bioactivity are combined. Such matrices can provide the needed structure as well as provide the needed signaling to recruit the required cells for OC TE. This would help achieve OC TE without utilizing cells and growth factors, an *in-situ* TE strategy to approach complex tissue, such as bone-cartilage interface.

In this study, decellularized cartilage ECM (dcECM) based bioinks were developed through an in-house developed rapid decellularization method [11]. The cartilage ECM was combined with viscosity modifiers and assessed for their rheological behavior and printability. The innovative aspect of this study is to identify spreadable ECM bioinks that could be used to achieve a continuous cartilage layer formation in OC grafts. The bioinks were also cellularized and their effects on the cellular behavior were assessed *in vitro*. dcECM matrices are capable of providing biochemical and biophysical properties of the native tissue through retention of many of the ECM components, such as collagen, proteoglycans and a reservoir of growth factors and cytokines. ECM biomaterials are involved in regulation of cellular behavior such as proliferation, differentiation and more recently has shown to possess chemotactic potential by directing cellular recruitment *in vitro* [11, 12, 23]. Therefore, we hypothesize that the decellularized ECM-based bioink will provide the native ECM-like composition and will enhance the chondrogenic activity and differentiation of encapsulated chondrocytes and bMSCs in a GF-free approach and the spreadable nature of the ECM bio-ink would allow continuous cartilage layer formation. Furthermore, we hypothesize that the developed bioink will be able to subsequently be used as part of an OC scaffold intended to support the cartilaginous tissue formation.

## 2. Materials and methods

### 2.1. Decellularization of cartilage tissue

Fresh bovine knee joints were obtained from Animal Technologies, TX (animals of age less than 30 months). Articular cartilage slices were isolated from the joints and decellularized under aseptic condition, as previously described [11]. Briefly, minced cartilage pieces were placed into a hypotonic Tris- HCl buffer solution (10 mM Tris-HCl, pH 8.0) and underwent six cycles of freezing (at −80 °C) and thawing (at 37 °C). The tissue was then treated with 0.25% trypsin solution for 1 h at 37 °C with vigorous agitation. After trypsinization, cartilage tissue pieces were washed with a hypertonic buffer solution (1.5 M NaCl in 50 mM Tris-HCL, pH 7.6) and treated with nuclease solution (50 U ml^−1^ DNAse and 1 U ml^−1^ RNAse A in 10 mM Tris–HCl, pH 7.5) with gentle agitation at 37 °C for 4 h. To remove all the enzymes, the enzyme-treated cartilage slurry was washed with the hypotonic Tris–HCL solution for 5 h, with fresh hypotonic solution replacements every 30 mins. After the nuclease treatment, tissue was treated with 1% Triton X-100 solution for 1 h. Lastly, the decellularized cartilage tissue pieces were washed with distilled water for 6 h to remove all the detergent, fresh water was replaced every 30 min. The processed tissue was then lyophilized and stored at −20 °C until further use.

### 2.2. Decellularized cartilage hydrogel preparation

dcECM was solubilized and converted into a gel, as previously described [24]. Briefly, the lyophilized cartilage tissue pieces were ground into a powder using a micro-mill grinder (Bel-Art, H37252). The powder was then suspended at 10 mg ml^−1^ in a 0.01 M HCl solution containing 2 mg ml^−1^ pepsin for 72 h at room temperature under vigorous agitation. To remove undigested particles, the solution was then centrifuged and the supernatant was collected. The solubilized cartilage ECM was combined with viscosity modifiers, xanthan gum (XG, Sigma-Aldrich) and nanoclay Laponite-XLG^®^ (BYK Additives & Instruments). XG and Laponite^®^ powders were pre-dissolved in DI water to form a homogenous gel. Then the solubilized dcECM was combined with the viscosity modifiers. The final concentrations of the hydrogels were 0.5% dcECM (%w/v) with varying concentrations of XG (0.5%, 1% and 1.5%) or 0.5% dcECM with varying concentrations of Laponite^®^ (2%, 3%, and 4%). The final hydrogels were thoroughly mixed and neutralized.

### 2.3. Rheological measurements

To determine printability, both steady shear and oscillatory shear experiments were performed using an AR-G2 rheometer (TA Instruments, DE) equipped with the serrated parallel plate fixture (diameter of 40 mm). In steady shear experiments, the apparent viscosity of the hydrogel samples was measured as the (maximum) shear rate increased from 0.01 to 10 s^−1^. In oscillatory shear experiments, strain sweeps were conducted as the strain amplitude increased from 0.01% to 1000% at a fixed frequency of 1 Hz. The linear viscoelasticity regime, storage modulus (G′), loss modulus (G″), loss tangent, and flow stress were subsequently identified. All studies were performed in triplicate at 37 °C.

### 2.4. Bioink printability

An extrusion-based bioprinter, BioX (CELLINK AB), was used for the printing of the dcECM-based bioinks, using a 25 G conical nozzle at room temperature with printing pressures 15–25 kPa. Testing of the bioink printability was conducted for the cartilage ECM gel combinations containing different viscosity modifiers. The printing was done in a square model (20 × 20 mm) with a rectilinear pattern and 20% infill density. Images of the printed constructs were analyzed using ImageJ software to quantify the print linewidth and spread ratio (SR) to analyze the overall printability of the cartilage ECM gel bioinks.

### 2.5. Bioink stability and degradation

To produce a stable bioink capable of photo-crosslinking, the bioinks were combined with a photo-curable PEG-DA (Sigma Aldrich) and photoinitiator, lithium phenyl-2,4,6-trimethylbenzoylphosphinate (LAP; CELLINK). The final bioink concentrations chosen were 0.5% dcECM, 2% Laponite^®^, 4% PEG-DA and 0.4% LAP. The dcECM bioink was loaded into a 3 ml cartridge and used for extrusion-based droplet printing on the BioX. The droplets were then exposed to 405 nm irradiation for 45 s to induce crosslinking, suspended in PBS and monitored for up to 21 d. Control samples were also formed without UV crosslinking to determine the effect of UV crosslinking on the stability of the bioink. Additionally, degradation of the bioink droplets was also monitored weekly up to 12 weeks. Briefly, bioink droplets were incubated at 37 °C in 1 ml of PBS. At pre-determined time points, samples were rinsed with deionized water, lyophilized, and weighed (*n* = 3). The remaining weight % was calculated by the following equation:

$$\%\text{Remaining}=\frac{Wt}{Wo}\times 100\%,$$

where Wo is the initial weight and Wt is the weight after time t.

### 2.6. Cartilage inks for growth factor loading and release

To test and model growth factor loading, bioink formulations were prepared and loaded with a model protein, bovine serum albumin which is fluorescently tagged with fluorescein (BSA-FITC). The loading concentration of 0.1 mg ml^−1^ was chosen as per our previous studies [21]. The fluorescently tagged BSA release was determined using a microplate reader (Synergy HT; BioTek Instruments) by reading the absorbance at 495 nm. The BSA-FITC loaded samples were also visualized using a fluorescent microscope (IX83, Olympus) with 494/517 nm excitation/emission to visualize the protein presence in the cartilage ECM bioink.

To determine the release of growth factor, the dcECM-based bioinks were loaded with PDGF-bb at a final concentration of 200 ng ml^−1^. A total of 10 droplets/samples of the final bioink was produced, crosslinked, and placed into a conical tube containing 15 ml of PBS at 37 °C. Supernatant was collected at pre-determined time-points and stored at −20 °C while replacing an equal amount of fresh PBS (300ul) daily. The GF release was determined using an Enzyme-Linked Immunosorbent Assay (*n* = 3).

### 2.7. Primary cell isolation

#### 2.7.1. Human MSC isolation

Human bone marrow-derived stromal cells (hBMSCs) were isolated from commercially available human bone marrow aspirate (BMA) (Lonza; donor: 24 year old female) using the lymphoprep density gradient method, as previously described [11, 25, 26]. Briefly, fresh human BMA was added directly to the lymphoprep solution and centrifuged at 1000 RPM for 20 min at room temperature. The mononuclear cell fraction was collected, resuspended in growth media (DMEM/F12 supplemented with 10% FBS (v/v), 1% penicillin/streptomycin (v/v)), and seeded onto tissue culture plates. Cells were cultured for 4 d at 37 °C in a 5% CO_2_ humid environment after which the non-adherent portion was washed away with gentle PBS washings. Adherent cells continued to be expanded to passage 2, with media change every 2–3 d and then frozen until further use. The human bone marrow derived MSCs of passage 2–3 cells were used for all studies.

#### 2.7.2. Rabbit articular chondrocyte isolation

Primary rabbit articular chondrocytes were isolated from rabbit femoral condyles. Briefly, freshly isolated rabbit knees were purchased (age 12–15 weeks) (Animal Technologies, Inc) and the articular cartilage from the femoral condyles were removed. The isolated cartilage was washed with PBS thoroughly and immersed in 20 ml of sterile Dulbecco’s Modified Eagle Medium/Ham’s F12 cell culture media (DMEM/F-12; Life Technologies) supplemented with 0.2% collagenase type II (ThermoFisher Scientific) and 1% penicillin/streptomycin (P/S; Life Technologies). The cartilage and collagenase supplemented media solution was then incubated in a 37 °C incubator with 5% CO_2_ and >98% humidity (HeracellTM VIOS 160i; ThermoFisher Scientific) for 12 h. The digest was then strained through a cell strainer with 70 μm sized pores, the flow-through was collected, and combined with DMEM/F- 12 supplemented with 10% fetal bovine serum (FBS; Life Technologies) and 1% P/S and their viability was confirmed. The cells of passage 2–5 were used for all studies.

### 2.8. Cartilage ink *in vitro* evaluation

To prepare the cell-laden bioinks, the dcECM-based bioink was prepared as previously described and termed ECM bioink (final formulation: 0.5% dcECM, 2% Laponite^®^, 4% PEG-DA and 0.4% LAP). Bioinks without the dcECM component were also prepared and termed non-ECM bioink (final formulation: 2% Laponite^®^, 4% PEG-DA and 0.4% LAP). Bioink components were syringe filtered for sterilization prior to cell encapsulation and prepared under sterile conditions. hBMSCs and chondrocytes were mixed with the bioinks at a final cell concentration of 10 × 10^6^ cells ml^−1^ by gentle mixing to prevent shear-induced cell death. Droplets were formed (containing 400 k cells) and crosslinked using 405 nm UV for 45 s. Chondrocytes were cultured in basal media (Dulbecco’s Modified Eagle Medium with GlutaMAX (DMEM + GlutaMAX; Life Technologies) supplemented with 10% fetal bovine serum and 1% penicillin-streptomycin (Life Technologies). On the other hand, hBMSCs were cultured in two different culture conditions, basal media (Dulbecco’s Modified Eagle Medium with Ham’s F12 with GlutaMAX (DMEM/F12 + GlutaMAX; Life Technologies) supplemented with 10% fetal bovine serum and 1% penicillin-streptomycin (Life Technologies)) and chondrogenic media (DMEM with GlutaMAX supplemented with ITS+, 100 μl ml^−1^ sodium pyruvate, 40 μg ml^−1^ L-proline, 50 μg ml^−1^ ascorbate-2-phosphate, 10^−7^ M dexamethasone, and 10 ng ml^−1^ TGF-b1) [27].

To assess cell viability, the cell-laden bioink droplets were subjected to Live/Dead staining after 1, 3, 7 d of culture. Live/Dead staining was conducted using LIVE/DEAD Viability/Cytotoxicity Kit (Thermo Fisher, MA) according to the manufacturer’s protocols. The stained samples were imaged using a fluorescent microscope with 494/517 nm excitation/emission for calcein-AM (green) for live cells and 528/617 nm excitation/emission for ethidium homodimer-1 (red) for dead cells.

To quantify the proliferation of cell-laden bioink droplets, the dsDNA concentration of each sample was measured using Quant-iT^™^ PicoGreen^™^ dsDNA Assay Kit (Thermo Fisher, MA). The samples were removed from culture after 1, 7, 14, and 21 d (*n* = 3), and lysed using 1% Triton X-100 and repeated freeze-thaw cycles. Afterward, the sample fluorescence was measured at 485/535 nm excitation/emission using a Synergy HT plate reader (BioTek Instruments, VT).

To quantify the chondrogenic activity of the cell-laden droplets, DMMB GAG quantification assay was conducted to measure the amount of chondrogenic ECM produced by the cells. First, samples after 7, 14, and 21 d of culture (*n* = 3) were removed and digested with a 1 mg ml^−1^ proteinase K solution for 16 h at 56 °C. The digest was then combined with a DMMB solution (0.016 mg ml^−1^) and its absorbance was measured at 520 nm (BioTek Instruments, VT). The results were then normalized using the corresponding DNA amounts obtained through PicoGreen Assay.

### 2.9. Histology

For qualitative assessment of the ECM composition, histological staining of the cell-laden droplets were conducted. The samples were removed after 7 d and 21 d of culture and fixed in 10% neutral buffered formalin (Fisher Scientific) for 1 h. The samples were then washed with PBS and embedded in HistoGel Specimen Processing Gel (Epredia) to protect the samples during processing for paraffin embedding. Followed by washing, the samples were sequentially dehydrated with increasing concentrations of ethanol and equilibrated with xylene. The samples were embedded in Histoplast PE paraffin (Fisher Scientific), sectioned at 5 μm using a Leica RM 2125 RTS microtome, and mounted on glass slides. Prepared slides were then deparaffinized and stained with Alcian Blue (Sigma-Aldrich) counterstained with Nuclear Fast Red (R&D Systems). The stained slides were then cover-slipped and imaged using brightfield microscopy (IX83, Olympus).

### 2.10. Statistical analysis

Quantitative data was reported as mean ± standard deviation. A one-way or two-way analysis of variance was used where appropriate, with Tukey’s *post-hoc* test at a significance level of *p* < 0.05 in all the statistical tests performed. *P < 0.05, **P < 0.01, ***P < 0.001, ****P < 0.0001

## 3. Results

**Figure 1.**
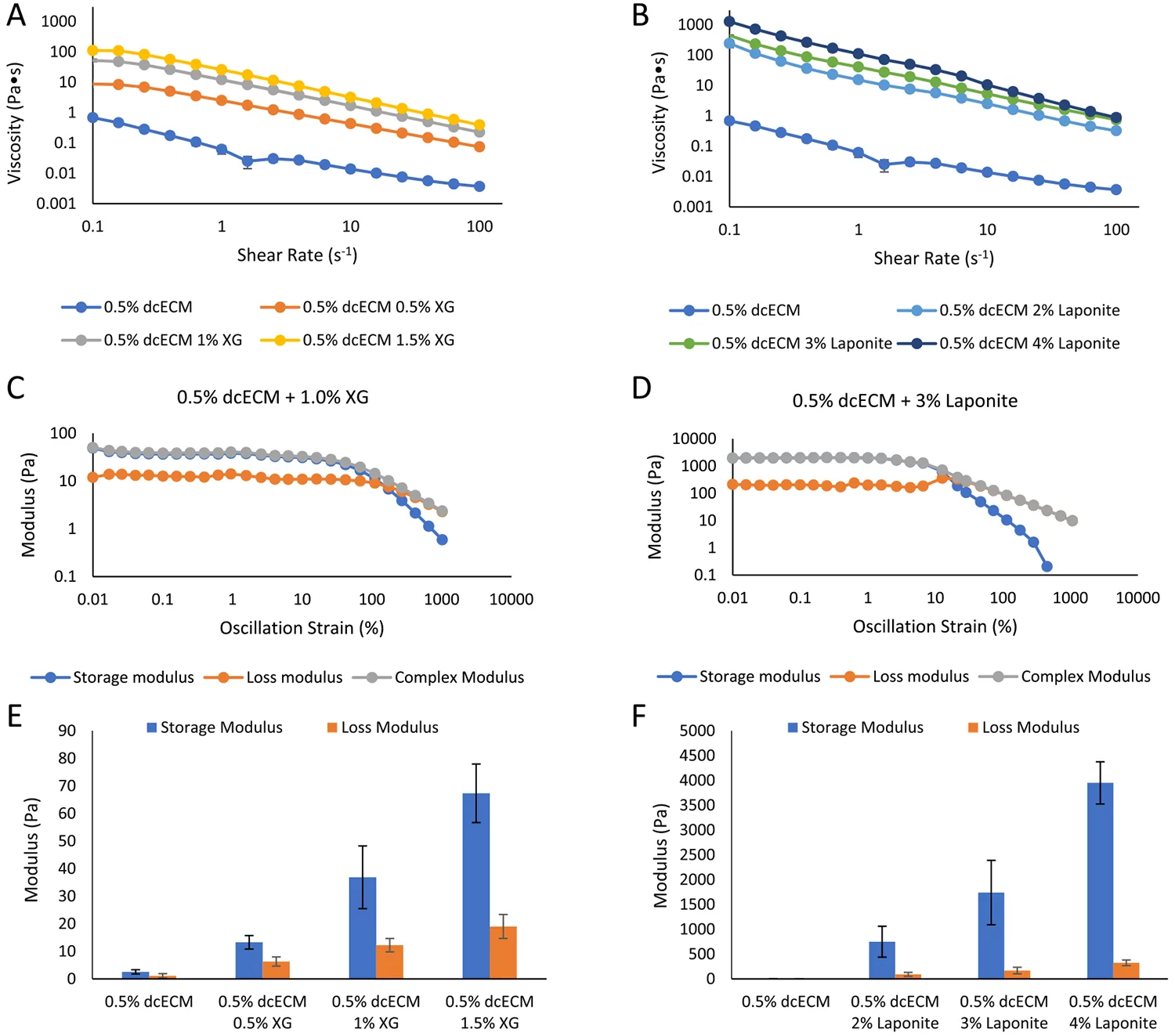
Rheological characterization of decellularized cartilage ECM (dcECM) bioinks. Viscosity vs. shear rate curves of (A) dcECM and dcECM-XG bioinks or (B) dcECM and dcECM-Laponite^®^ bioinks, demonstrating shear-thinning behavior and varied viscosity of each formulation (*n* = 3). Representative oscillatory strain sweeps conducted on (C) dcECM-XG and (D) dcECM-Laponite^®^ bioinks. Storage (G′) and loss (G′’) moduli of (E) dcECM and dcECM-XG bioinks or (F) dcECM and dcECM-Laponite^®^ bioinks, within the linear viscoelasticity regime (*n* = 3). Formulations exhibited an increase in moduli with increasing concentration of each viscosity modifier.

To increase the viscosity of the decellularized cartilage bioink for extrusion-based bioprinting, the solubilized dcECM was combined with viscosity modifiers XG and Laponite^®^. Figures 1(A) and (B) show the flow curves of a 0.5% dcECM solution containing different amounts of XG and Laponite^®^, respectively. All samples, including the base 0.5% dcECM solution, exhibited shear thinning, where the viscosity decreased as a function of increasing shear rates. Shear thinning behavior is desirable for extrusion-based bioprinting because the shear deformation imposed on the bioink during printing will reduce the viscosity and consequently lower the extrusion pressure. As the ink is deposited on a substrate, the shear rate diminishes, and a higher viscosity at low shear rates will improve shape retention [28-30]. In both cases of XG and Laponite^®^, the overall viscosity increased as the viscosity modifier concentration increased and exhibited a power-law dependence on shear rates. The degree of shear thinning may be further quantified by the flow index (*n*′) from power-law fitting to the viscosity data ($\eta_{a}=K{\dot{\gamma }}^{n^{\prime }-1}$), as summarized in table 1. The lower the *n*′ value, the higher the degree of shear thinning. The *n*′ value and degree of shear thinning for both XG and Laponite^®^ samples showed dependence on the viscosity modifier concentration, in which a higher concentration of the viscosity led to a higher degree of shear thinning. Further, the dcECM-XG solutions have a *n*′ values of 0.14–0.27, whereas dcECM-Laponite^®^ formulations have *n*’ values of −0.04 to 0.1. Figures 1(C) and (D) showed the storage modulus (G′), loss moduli (G″), and magnitude of the complex modulus ($|G^{*}|$) as a function of strain amplitude for 1% XG and 3% Laponite^®^, respectively. First, in both samples, G′ dominated over G″ at low strains, consistent with their behavior as soft solids at rest. However, the Laponite^®^ sample showed a higher $|G^{*}|$ at low strains, which is desirable for shape retention. Second, the crossover between G′ and G″ ($\sigma_{f}$) signifies the solid–liquid transition, or the flow stress, as the sample undergoes deformation during printing [31-33]. Despite the higher moduli at low strains, $\sigma_{f}$ (ca. 17.09%) of the Laponite^®^ sample is about an order magnitude smaller than that of the XG sample (ca. 142.32%), implying that the Laponite^®^ sample will start to flow under a smaller deformation and is thus easier to extrude. Figures 1(E) and (F) showed the viscosity modifier concentration’s effects on the G′, and G″. For both viscosity modifiers, the increase in G′ is larger than the increase in G″ as the modifier concentration increases. The loss tangent ($\tan(\delta )=G^{\prime \prime }∕G^{\prime }$)) was calculated to decrease from 0.470 to 0.282 for dcECM-XG and from 0.122 to 0.082 for dcECM-Laponite^®^ as the viscosity modifier concentration increased. These results indicate that the dcECM-Laponite^®^ formulations are more solid-like compared to the dcECM-XG formulations. Within the linear viscoelasticity regime, the dcECM-Laponite^®^ samples have a G′ ranging from 750 to 4000 Pa, which is three orders of magnitude higher than that for the dcECM-XG samples ranging from 13 to 67 Pa. Overall, the dcECM-Laponite^®^ samples have a considerably higher stiffness compared to the dcECM-XG samples for the concentration ranges considered.

**Figure 2.**
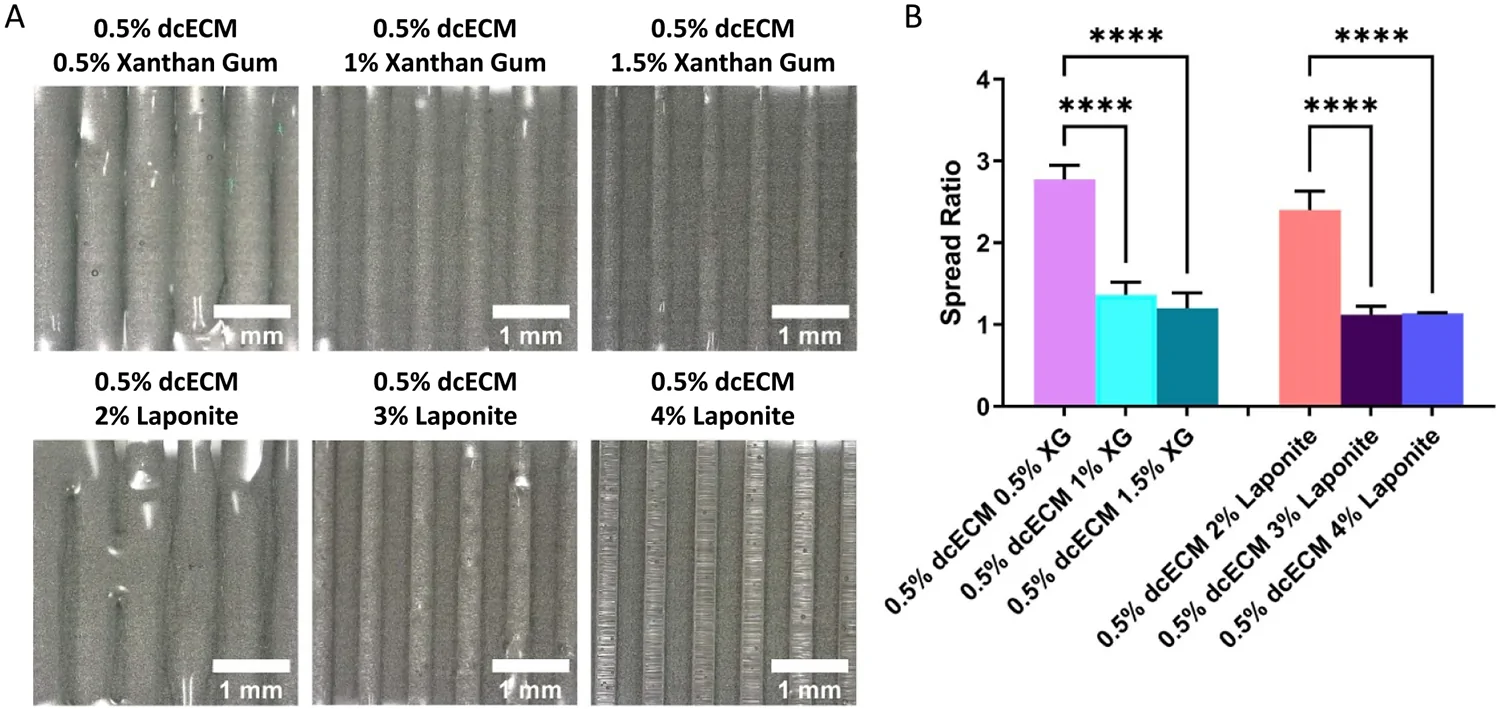
Printability of cartilage ECM bioinks. (A) Representative images of single layer printed dcECM-based bioinks with varying viscosity modifiers and their concentrations. (B) Calculated spread ratio of the dcECM-based bioinks, showing the trend with respect to viscosity modifier and concentration. Asterisks denote significance of ****P < 0.0001.

Furthermore, the propensity for dcECM-XG samples and dcECM-Laponite^®^ samples to retain shape after printing was compared based on the SR, which is defined as: SR = extrudate diameter/nozzle diameter [34, 35]. A SR close to 1 suggests the printed line matches the diameter of the print needle. Figure 2(A) shows the images of the printed lines for different XG and Laponite^®^ concentrations. The linewidth clearly decreased as the XG concentration increased from 0.5% to 1%. Similar results were also observed as Laponite^®^ concentration increased from 2% to 3%. However, increasing the XG or Laponite^®^ concentration further did not reduce the SR as the printed linewidth already approached the needle diameter. The lower SR of the printed lines for samples with a higher viscosity modifier concentration is consistent with the higher viscosity of these samples, which slows down the spreading and/or lead to a smaller volumetric flow rate. For the same nozzle translation speed, a smaller volumetric flow rate will also lead to a smaller footprint [32, 33]. Another possible explanation is that the XG and Laponite^®^ may have increased the contact angle, leading to a smaller footprint. The SR of XG-and Laponite^®^-modified inks were comparable for a single-layer print, but the Laponite^®^-based formulation was chosen over the XG-one given their higher moduli, which is important for sustaining multi-layer structures, and their solid-fluid transition at a smaller strain amplitude (i.e. smaller $\sigma_{f}$). Together, the data demonstrates the tunability of the viscosity of the bioinks and shear thinning properties, allowing the formulations to be readily extruded and maintain shape fidelity of the structure post-printing.

**Figure 3.**
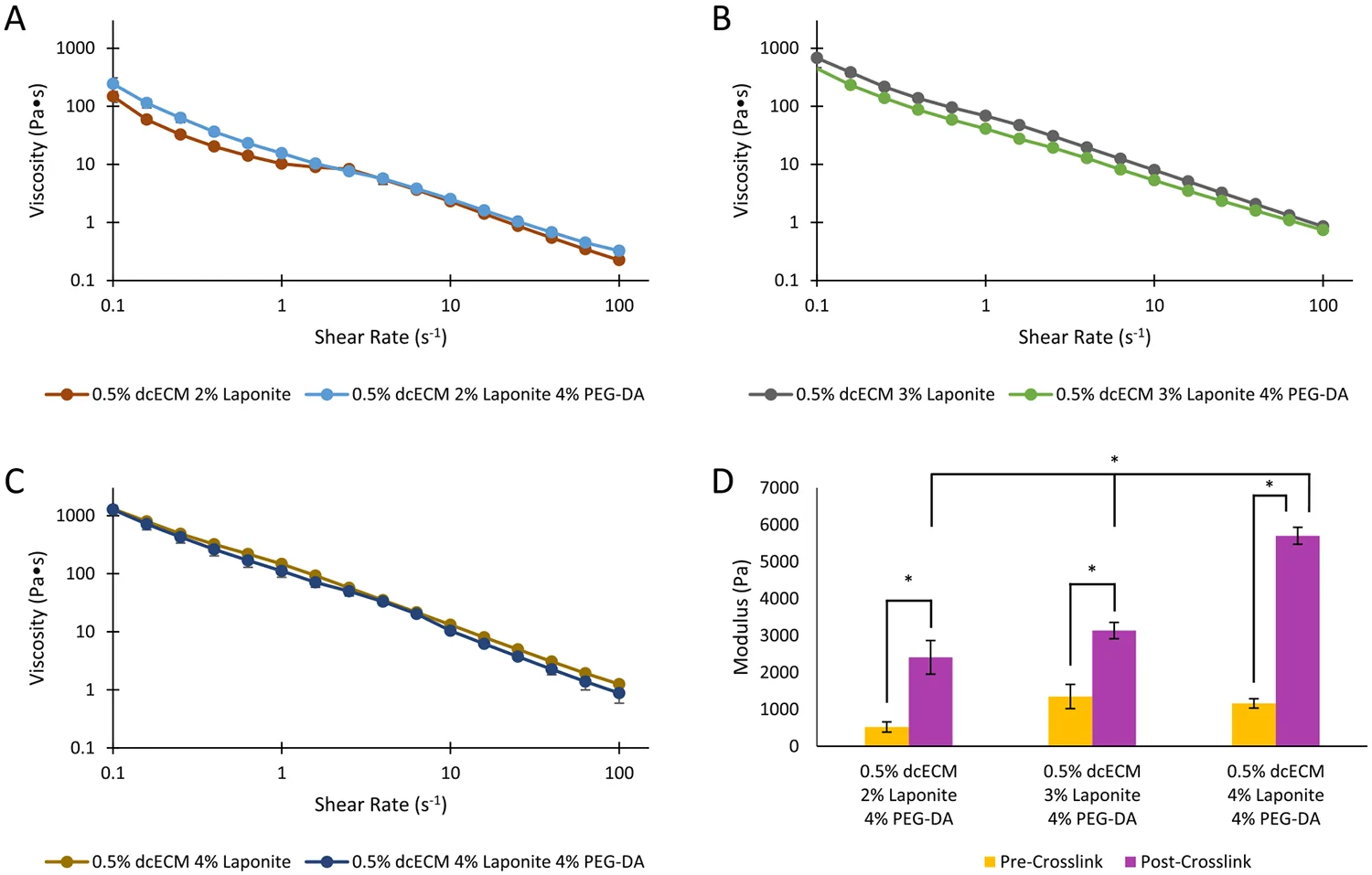
Rheological characterization of photo-crosslinkable cartilage ECM bioinks. (A)–(C) Viscosity vs. shear rate curves of dcECM-Laponite^®^ and photo-crosslinkable dcECM-Laponite^®^ formulations with different concentrations of Laponite^®^, prior to crosslinking. Photo-crosslinkable formulations demonstrated shear-thinning behavior comparable to non-crosslinkable formulations. (D) Storage moduli of photo-crosslinkable dcECM-Laponite^®^ formulations before and after UV crosslinking. Cross-linked bioink formulations exhibited a significant increase in moduli. Asterisks denote significance of *P* < 0.05.

Additionally, solubilized dcECM derived from pepsin-digestion can undergo thermal gelation at physiological conditions (37 °C temperature and neutral pH). However, they typically possess poor mechanical properties and are easily susceptible to enzymatic and *in vivo* degradation. To improve gel stability, dcECM can be modified by the addition of methacrylate groups or combined with a secondary component that is capable of photo-crosslinking in the presence of a photo-initiator. In this study, PEG-DA was utilized in combination of the dcECM/Laponite^®^ formulations. Steady shear testing of the bioinks were performed on dcECM/Laponite^®^ with or without the PEG-DA. The results showed that the incorporation of the PEG-DA did not affect the flow behavior of the bioinks, wherein the bioinks showed similar viscosity values and exhibiting shear-thinning behavior (figures 3(A)-(C)). However, UV crosslinking of the dcECM/Laponite^®^ bioinks demonstrated differences in their storage moduli values (Figure 3(D)). Crosslinking of the bioink formulations showed a significant increase in their storage moduli from approximately 525–2400 Pa for the lower Laponite^®^ concentration, 1350–3130 Pa for the intermediate Laponite^®^ concentration and 1160–5700 Pa for the high concentration of Laponite^®^. The incorporation of both XG and Laponite^®^ improved the rheological and printability properties of the dcECM-based bioinks. Moreover, based on the lower viscosity and higher SR of the lower concentration of Laponite^®^, 0.5% dcECM/2% Laponite^®^/4% PEG-DA bioink was selected as being sufficient to impart the desired rheological properties, possess the ability to spread to appropriately infuse into a 3D template while reducing shear stresses induced during printing to maintain cell viability.

**Figure 4.**
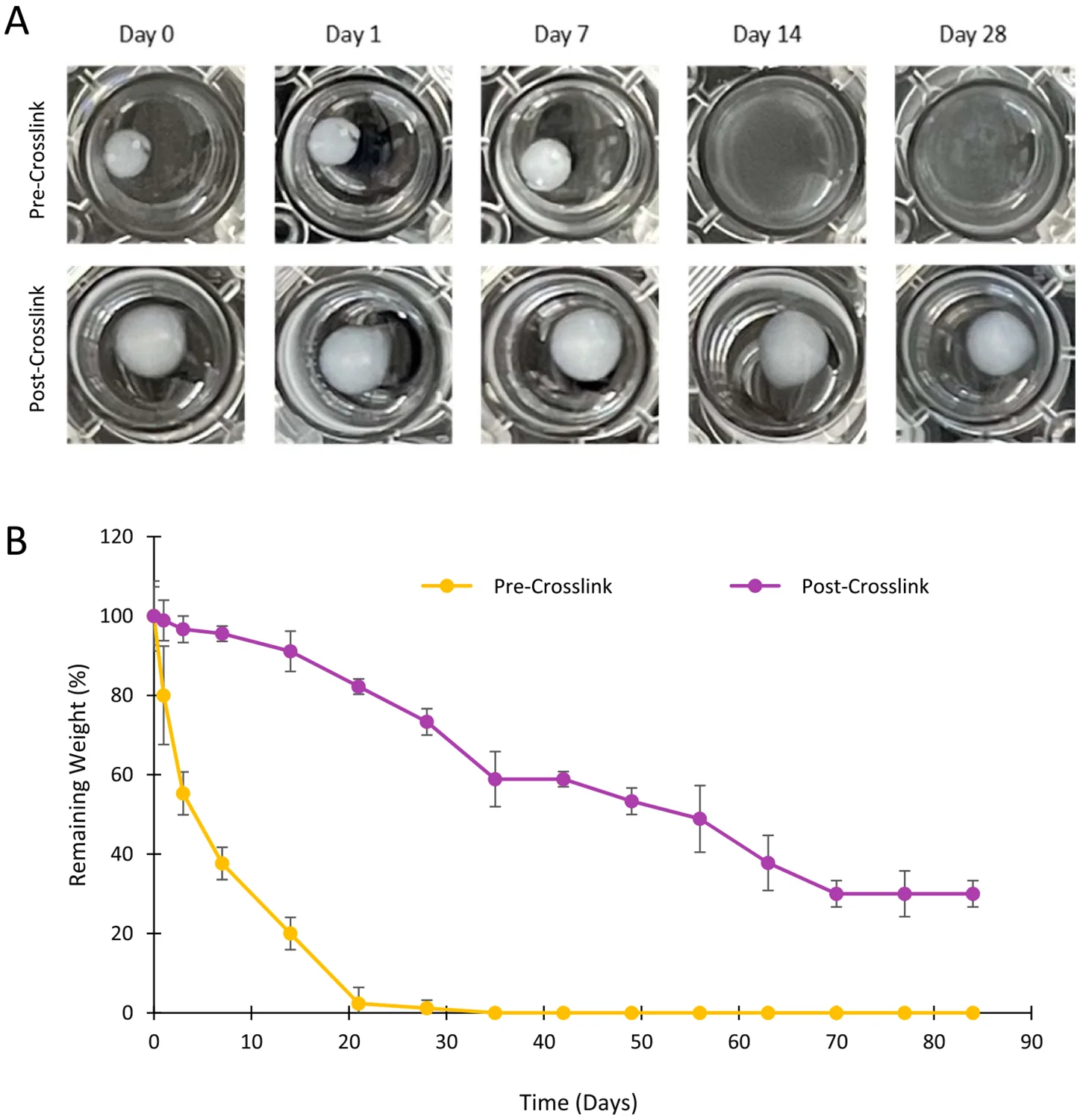
Stability and degradation of decellularized cartilage ECM (dcECM) bioinks. (A) Representative images of dcECM-based bioinks without or with UV-crosslinking, and (B) degradation patterns (*n* = 3). Bioink droplets without UV-crosslinking (pre-UV) demonstrated limited stability and rapid degradation, whereas UV crosslinked droplets (post-UV) showed increased stability with controlled degradation.

Furthermore, the bioink stability was also assessed by culturing the bio-printed droplets in PBS at 37 °C. For this study, the bioink droplets were divided into two groups: one group without UV-crosslinking (pre-UV) and the other group with UV-crosslinking (post-UV). The study demonstrated that pre-UV (thermal gelation only) droplets were only stable up to Day 7 (figure 4(A)). This was also observed in degradation study which demonstrates that there is a rapid decrease in overall mass associated with the pre-UV droplets (approximately 38% mass remaining by Day 7 and 20% by day 14 (figure 4(B))). However, post-UV, the bioinks formed stable droplets throughout the duration of the study and maintained integrity (up to day 28), as seen in figure 4(A). Degradation studies also demonstrated the gradual degradation associated with the post-UV droplets. Results showed approximately 80% mass remaining of the post-UV droplets by Day 21 and 30% mass remaining by Day 84. Therefore, it is clear from the degradation studies that UV-crosslinked bioink formulations showed improvements in their stability and degradation overtime, whereas the bioinks with thermal gelation only showed rapid degradation and complete elimination in less than three-weeks.

**Figure 5.**
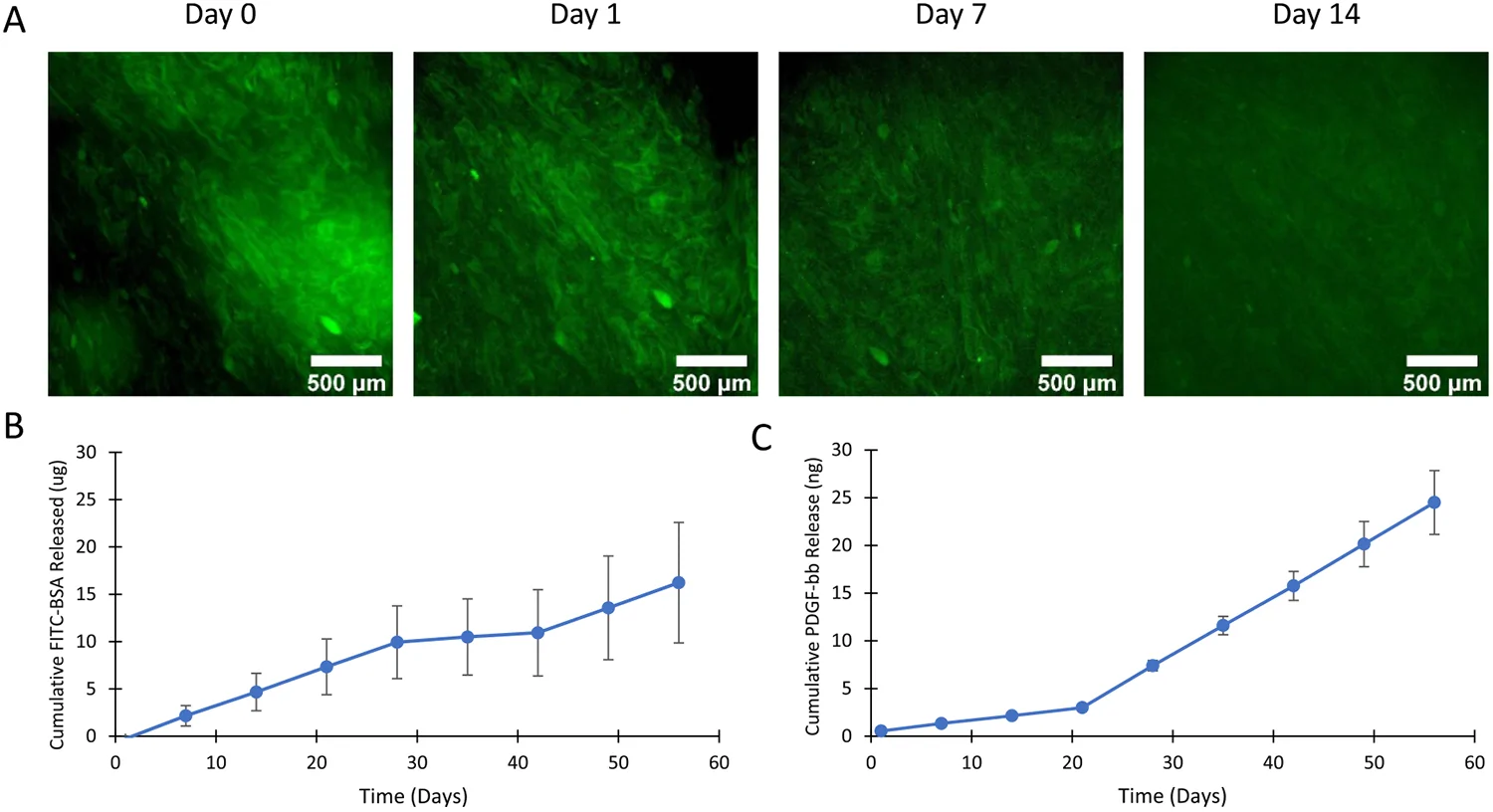
Model protein and growth factor loading and release from decellularized cartilage bioinks. (A) Fluorescent images of the dcECM-based bioink droplets loaded with FITC-BSA, the images were recorded on Day 0, 1, 7, and 14. Cumulative release of (B) FITC-BSA and (C) PDGF-bb from the UV-crosslinked bioink droplets (*n* = 3).

Laponite^®^, including its role and use as a viscosity modifier, has also been commonly used for protein and drug loading due to electrostatic interactions. Fluorescently labeled model protein was used to visualize growth factor loading and distribution in the bioink. The dcECM/Laponite^®^/PEG-DA bioink (post-UV) was prepared with FITC-conjugated bovine serum albumin (FITC-BSA) and visualized using fluorescent microscopy. Images demonstrate effective loading of the protein within the bioink on Day 0 (figure 5(A)). The presence of the loaded model protein was seen until Day 14. Release studies show the slow release of BSA-FITC with about a third of the estimated total protein loaded is released in 8 weeks (figure 5(B)). The bioink system was also loaded with a growth factor, PDGF-BB, and monitored for release. Similar to FITC-BSA, the growth factor also showed the slow release of PDGF-BB up to 8 weeks without bulk release. Moreover, cumulative release study demonstrated that the dcECM/Laponite^®^ bioink system (post-UV) allowed for slow release with nearly 75% of the loaded PDGF-bb remaining after 8 weeks (figure 5(C)). These results demonstrate bioink’s ability to incorporate proteins and growth factors, and further allow for their retention and sustained release.

**Figure 6.**
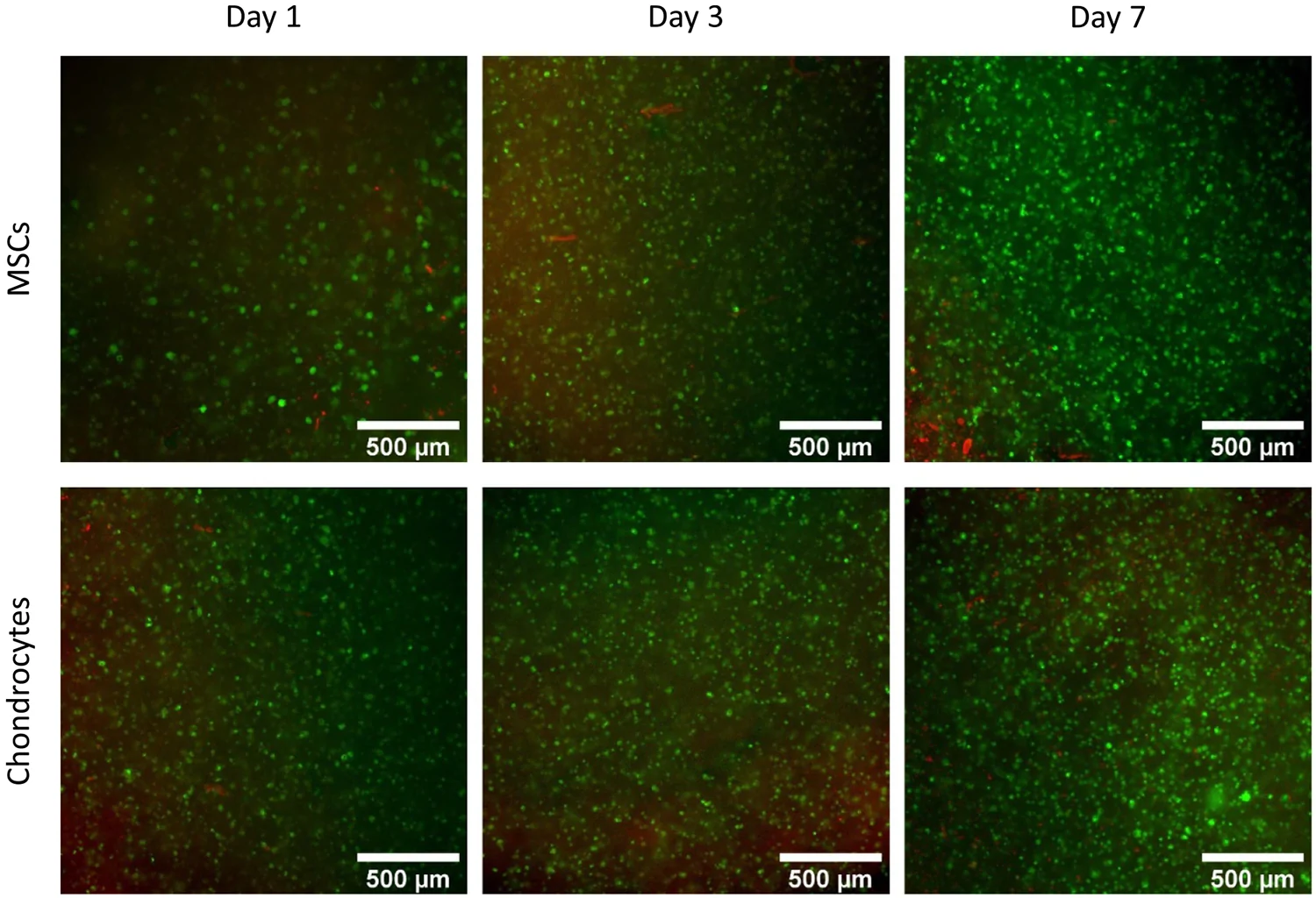
*In vitro* assessment of dcECM-based bioink biocompatibility. Live/Dead images of (A) hBMSCs or (B) chondrocytes encapsulated within the UV-crosslinked (post-UV) bioink on Day 1, 3, 7. Live/Dead images show mostly live cells with minimal cell death.

To determine whether dcECM/Laponite^®^/PEG-DA bioink could support cell viability, biocompatibility assessment was conducted *in vitro* through Live/Dead staining. The bioink was loaded with hBMSCs or chondrocytes, bio-printed to produce droplets, UV-crosslinked, and cultured for 7 d. As shown in figure 6(A), hBMSCs showed viable cells up to Day 7, with minimal cell death as evident by dominance of green color, therefore live cells. Moreover, it was shown that the hBMSCs also proliferated between Day 1 and 7 as is evident by the increase of green signal. Similar results were also observed for the chondrocyte-loaded bioink droplets (figure 6(B)). Live/dead staining of chondrocytes showed the prevalence of live cells, with minimal cell death which remained almost the same throughout the study.

**Figure 7.**
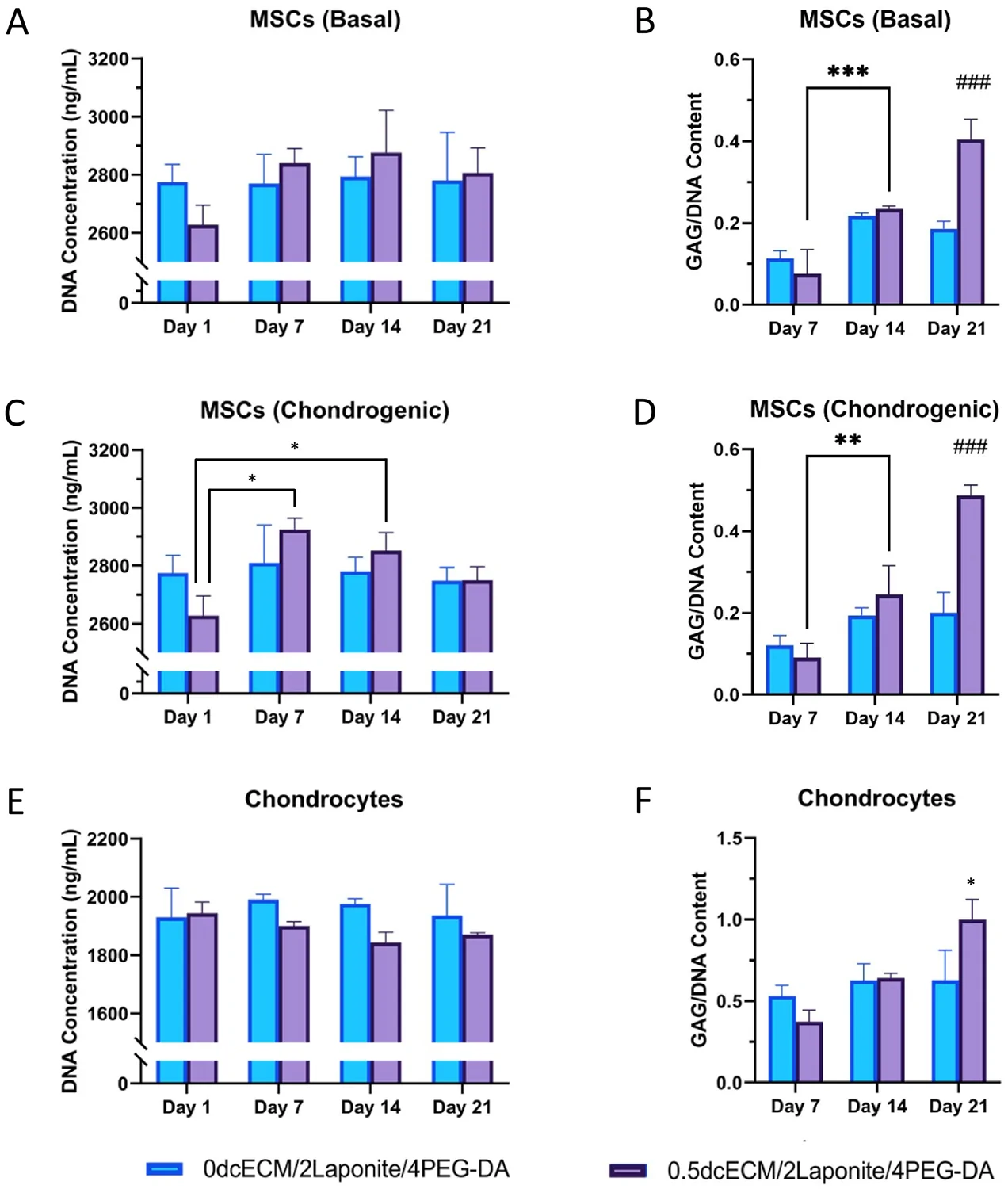
Cell proliferation and chondrogenic activity of the decellularized cartilage ECM (dcECM) bioinks. PicoGreen dsDNA quantification of hBMSCs encapsulated in dcECM bioink and cultured in (A) basal or (C) chondrogenic medium (*n* = 3) and (E) chondrocytes encapsulated in dcECM bioink after 1, 7, 14 and 21 d (*n* = 3). sGAG quantification of hBMSCs encapsulated in dcECM bioink and cultured in (B) basal or (D) chondrogenic medium and (F) chondrocytes encapsulated in dcECM bioink after 7, 14 and 21 d of culture. Asterisks denote significance of *P < 0.05, ** represents *P* < 0.05, *** *P* < 0.001, ### *P* < 0.001 when compared with ECM for days 7 & 14 and without ECM for day 21.

After confirming the basic viability of hBMSCs and chondrocytes, the dcECM/Laponite^®^/PEG-DA bioink was characterized for its ability to induce chondrogenesis *in vitro.* For hBMSCs, two different culture conditions were used, namely cultured in basal medium or chondrogenic medium (to prime hBMSCs towards the chondrogenic lineage), whereas terminally differentiated chondrocytes were also cultured in basal medium. Cellular proliferation and chondrogenic activity were assessed for cells cultured in 0.5dcECM/2Laponite^®^/4PEG-DA (containing decellularized ECM, ECM bioink) and compared to 0dcECM/2Laponite^®^/4PEG-DA (no decellularized ECM, non-ECM bioink), measured using PicoGreen dsDNA and DMMB sGAG quantification (figure 7). The non-ECM bioink encapsulating hBMSCs did not show any significant changes in DNA content throughout the duration of culture up to day 21, regardless of culture conditions (Figures 7(A) and (C)). In contrast, cartilage ECM containing bioink encapsulated with hBMSCs showed an increase in DNA content throughout the duration of the culture in basal medium, which peaked at Day 14 (figure 7(A)). When cultured in chondrogenic medium, the ECM containing bioink with hBMSCs showed a significant increase in DNA content up to Day 14, which peaked at Day 7, indicating increased levels of cellular proliferation (figure 7(C)). Additionally, ECM and non-ECM containing cartilage bioinks showed differences in their sGAG production as measured by DMMB assay. For non-ECM containing bioink, sGAG production slightly increased at Day 14 and leveled off at Day 21, regardless of culture condition (basal or chondrogenic) (figures 7(B) and (D)). The ECM containing bioink (basal), on the other hand, showed significantly higher levels of sGAG production after Day 7, which continued to Day 21 (figure 7(B)). This behavior of increased sGAG production for the ECM containing bioink was more pronounced for chondrogenic-medium primed hBMSCs, which showed higher levels of sGAG production at the intermediate time point (Day 14), compared to the non-ECM containing bioink and continued to Day 21 (figure 7(d)). Similar cell proliferation results were observed when chondrocytes were cultured in ECM containing and non-ECM containing bioinks. Both groups showed that the DNA content was maintained or slightly increased throughout the culture duration, indicating that the chondrocyte levels of proliferation were maintained or slightly increased (Figure 7(E)). However, the sGAG production of chondrocytes encapsulated in the ECM containing bioink was significantly increased compared to non-ECM containing group after 21 d of culture (figure 7(F)).

**Figure 8.**
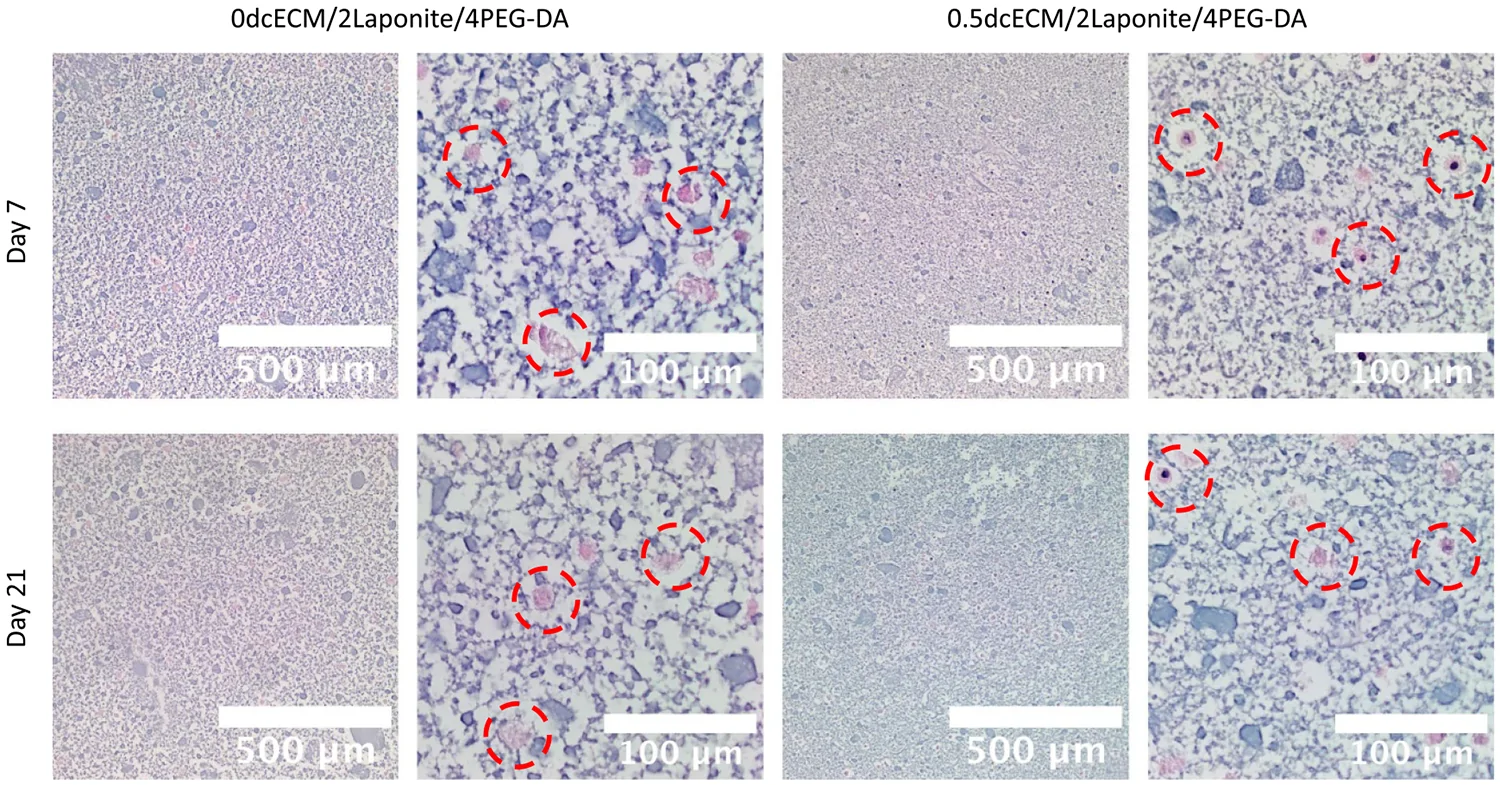
Histological assessment of the *in vitro* cultured cartilage bioinks with hBMSCs in basal media. Alcian blue staining of the encapsulated cells within the ECM-containing and non-ECM containing bioink formulations cultured in basal medium for 7 and 21 d. Representative images were recorded at 4× and 20× magnification. Example cells are highlighted with the red dotted circle.

**Figure 9.**
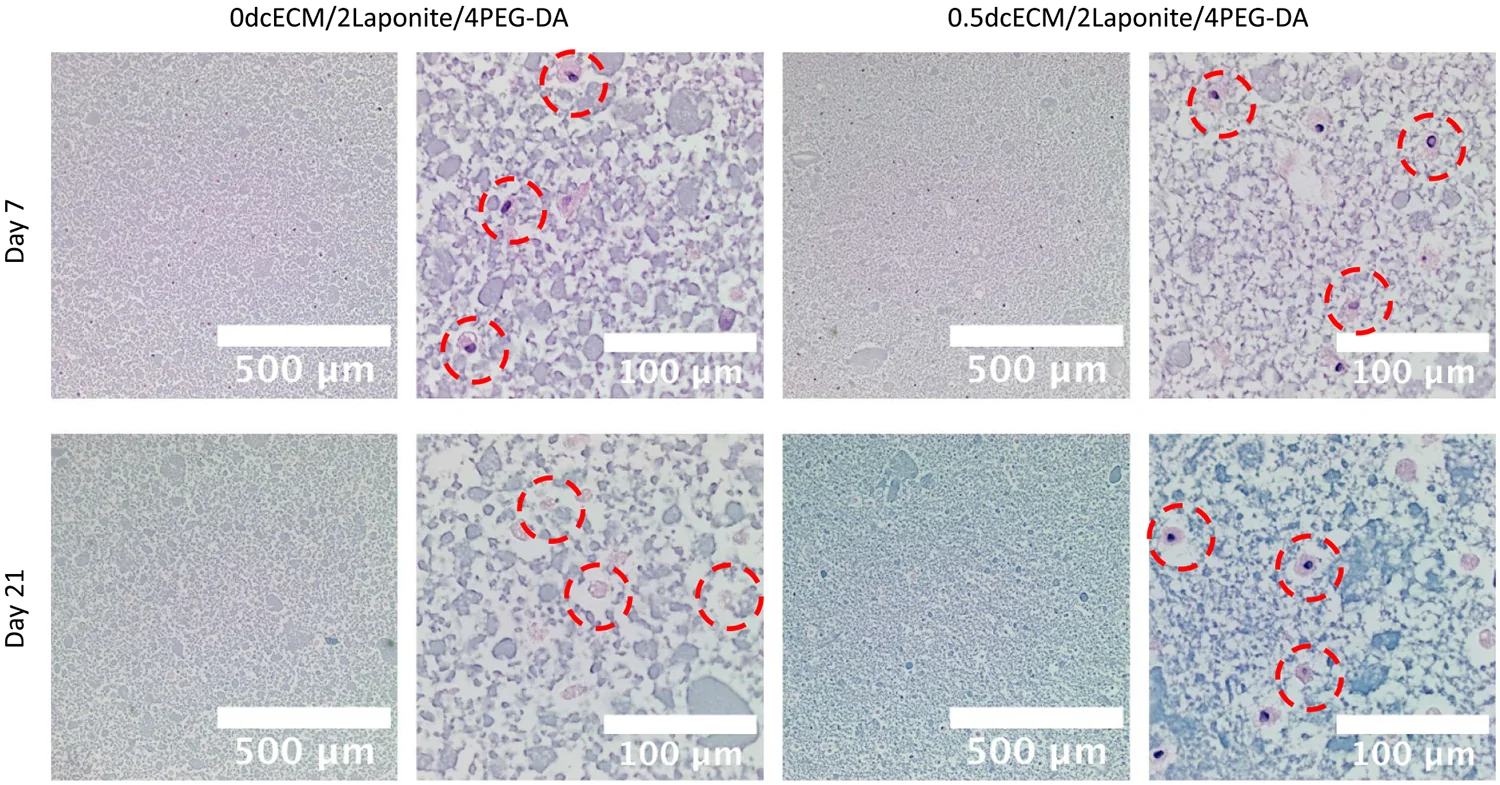
Histological assessment of the *in vitro* cultured cartilage bioinks with hMSCs in chondrogenic media. Alcian blue staining of the encapsulated cells within the ECM-containing and non-ECM containing bioink formulations cultured in chondrogenic medium for 7 and 21 d. Representative images were recorded at 4× and 20× magnification. Example cells are highlighted with the red dotted circle.

**Figure 10.**
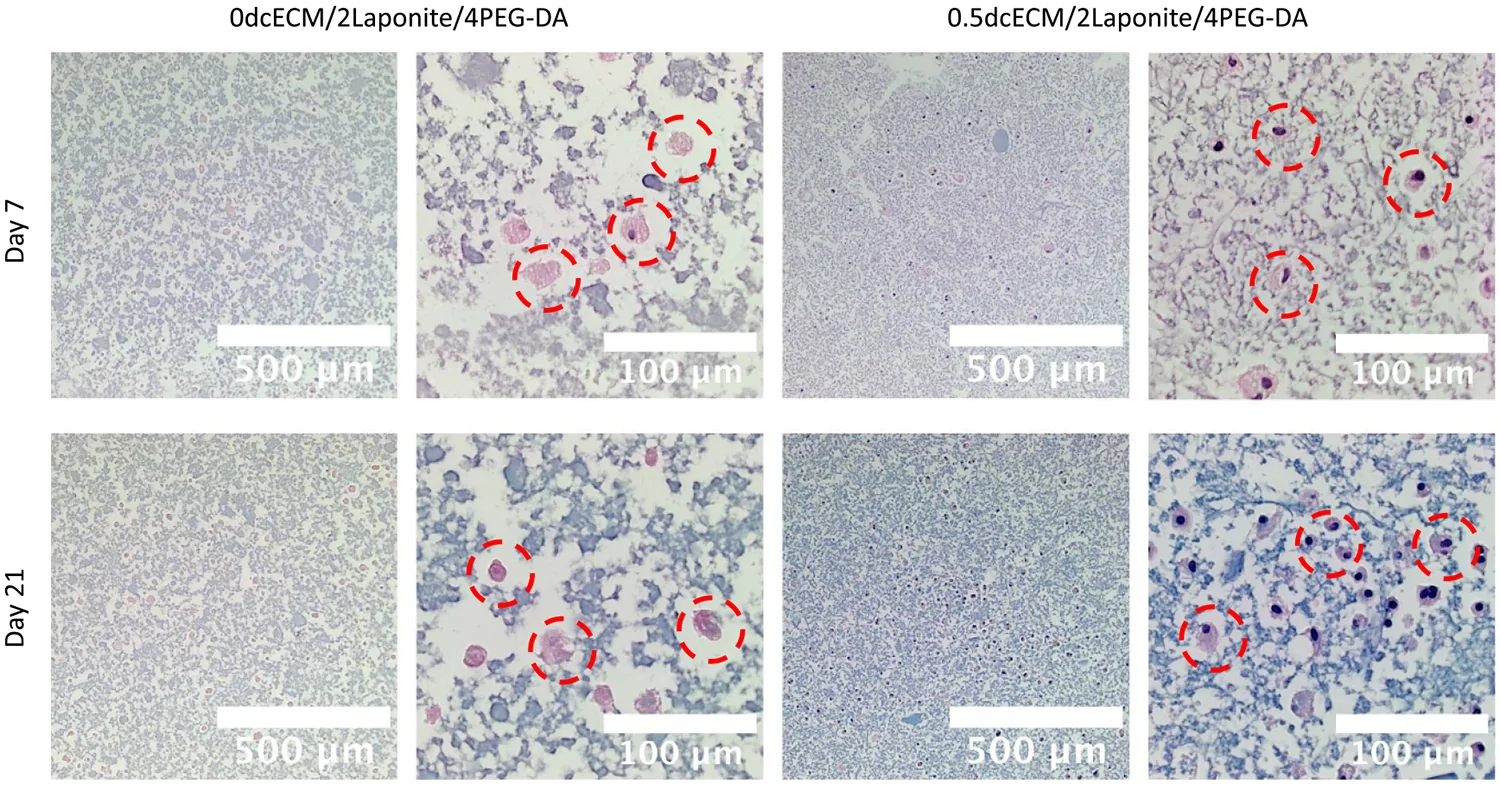
Histological assessment of the *in vitro* cultured cartilage bioinks with chondrocytes in basal media. Alcian blue staining of the encapsulated cells within the ECM-containing and non-ECM containing bioink formulations cultured in basal medium for 7 and 21 d. Representative images were recorded at 4× and 20× magnification. Example cells are highlighted with the red dotted circle.

To further evaluate the developed bioink chondrogenic activity *in vitro*, qualitative analysis through Alcian blue was performed. As shown in figures 8 and 9, non-ECM bioink encapsulated with hBMSCs showed minimal levels of ECM on Day 7, as evident by the lack of Alcian blue staining, regardless of culture (basal or chondrogenic). After 21 d of culture, the results for hBMSCs (basal) remained similar with little to no increase in the Alcian blue staining. For hBMSCs (chondrogenic), after 21 d of culture in non-ECM bioink, the results show very little visibly detectable Alcian blue staining, signifying the lack of GAG deposition by the encapsulated hBMSCs. Similarly, the ECM containing bioink showed minimal levels of GAG production at the early time point (Day 7), regardless of culture condition (basal or chondrogenic) (figures 8 and 9). This was in agreement with the quantitative sGAG results, which showed similar levels for all groups at Day 7. However, by Day 21 the hBMSCs (basal) showed increased levels of Alcian blue staining compared to non-ECM containing bioink (figure 8). Meanwhile, Alcian blue staining of hBMSCs (chondrogenic) showed more pronounced and higher levels of Alcian blue staining indicating enhanced sGAG production of the encapsulated hBMSCs and their chondrogenic activity (figure 9). For chondrocytes, the histological staining showed a similar trend with little to no amount of Alcian blue staining on Day 7, regardless of bioink formulation (figure 10). However, by Day 21 chondrocytes encapsulated in ECM bioink showed much greater amounts of Alcian blue staining in the matrix than the non-ECM bioink. Furthermore, it was also observed that the chondrocytes encapsulated in the ECM bioink are similar to native chondrocytes within the lacunae of the ECM such that they possess a round to oval/oblong shape [36]. The increased Alcian blue staining indicates the cartilage ECM containing bioink’s ability to promote MSC chondrogenic differentiation and maintenance of chondrocyte phenotype and chondrogenic activity.

## 4. Discussion

The decellularization of tissues and their use as bioinks can provide many benefits for the biofabrication of tissue analogs, including the preservation of native ECM-like features and components as well as the fabrication of precisely defined and controlled designs [13, 37]. However, bioinks derived from natural polymers and biomaterials can exhibit very low viscosity, and poor resistance to degradation [38, 39]. In this study, we developed bioactive cartilage tissue inks for cartilage TE or that can be combined with OC grafts for OC TE. The novelty of the cartilage tissue inks is the development of non-spreadable or spreadable tissue inks intended for a wide variety of applications. These include stackable 3D bioink-based scaffolds or the selective infusion of spreadable tissue inks within the desired zones of a 3D porous template, ability to concomitantly print cartilage layer in OC scaffolds, and flexibility to introduce cell type and cell density needed to form a continuous layer of articular cartilage in OC grafts.

In this study, the dcECM has been successfully processed into a solubilized form using an in-house developed rapid decellularization protocol [11]. However, the viscosity of the solubilized dcECM was too low for retaining the shape of the printed lines in extrusion-based printing. To that end, varying concentrations of two different viscosity modifiers, namely XG and Laponite^®^, were combined with the dcECM in efforts to increase the bioink viscosity. Inclusion of XG and Laponite^®^ increased the overall viscosity but also preserved the shear thinning behavior which is desirable for extrusion-based 3D printing [40]. The increase in viscosity can be attributed to the XG or Laponite^®^ associations in the bioink formulation [35]. In addition to shear viscosity, XG and Laponite^®^ also increased the stiffness of the bioink which is beneficial for sustaining the weight of upper layers in multi-layer printing. Under small deformations, both dcECM-XG and dcECM-Laponite^®^ bioinks behaved as an elastic soft solid with G′ dominating over G″. Despite the comparatively higher storage modulus of the dcECM-Laponite^®^ bioinks, the transition from solid-like to liquid-like behavior was observed at a smaller strain amplitude, implying that they are easier to be extruded compared to the dcECM-XG bioinks. Differences in the elastic modulus between the XG and Laponite^®^ groups could have arisen due to the differences in the concentrations as well as compositional variations between them [41, 42]. Compared to the solubilized dcECM samples without any viscosity modifier, the more viscous and stiffer bioink will ensure the stability of the printed bioink following extrusion, or in general for 3D printing [43].

The rheological effects can be directly observed in the printing performance of the bioink formulations as the degree of spreading of the strut width correlated with the concentration of the viscosity modifier. An increase in concentration led to continued improvement in the shape fidelity whereas low concentrations dramatically promoted strut spreading. This behavior demonstrates the capability of tailoring the bioink formulation for the desired application. By adjusting the concentration of the viscosity modifiers, different strategies can be developed. For example, the concentration of the viscosity modifier and its resulting shape fidelity post-printing can allow for stackable 3D bioink-based scaffolds (higher concentration range) or can allow for hybrid scaffold fabrication wherein a 3D porous template can be subsequently infused with the bioink (lower concentration range). The former has additional benefits wherein the utilization of higher concentration viscosity modifier may allow for high-fidelity extrusion of the bioink with increased mechanical properties, however, can ultimately affect cellular viability and may not be suitable for OC TE [44, 45]. Alternatively, the combination of a porous 3D template and infusion of the bioink can be employed to take advantage of the mechanical properties of the template while supporting viability and functionality. Our previous studies have developed such porous template scaffolds for OC interface engineering [22]. However, this study is focused on the development of a bioactive cartilage tissue ink for the cartilage zone of the OC grafts.

Bioink crosslinking is critical for the maintenance of the printed structures and to provide a microenvironment with proper mechanical properties and stability for cellular activities [46, 47]. While the ECM-based bioink can undergo thermal gelation at physiological conditions, the formation of a more stable network requires long gelation times which can negatively affect shape fidelity and cellular survival due to deprivation of essential nutrients as well as possess poor mechanical properties and are susceptible to degradation [48, 49]. Herein, the dcECM-Laponite^®^ bioinks were combined with a photo-crosslinkable component to allow for a stable network formation. The incorporation of the photo-crosslinkable component, PEG-DA, did not alter the shear-thinning behavior of the bioink formulations. However, upon exposure to UV irradiation, the bioink formulations demonstrated increased mechanical properties due to the formation of the polymeric network [50, 51]. Additionally, the increased mechanical properties of the higher concentration of Laponite^®^ are likely due to the increased contribution and possible electrostatic interactions between Laponite^®^ surfaces for the stability of the bioink through non-covalent interactions [52, 53]. However, the higher modulus corresponding to increased matrix stiffness may provide a restrictive microenvironment to encapsulated cells, limiting nutrient and oxygen diffusion, affect the degree of cell-cell/cell-ECM interaction, and ECM remodeling and therefore the lower Laponite^®^ concentration formulation was selected [54-56]. The introduction of the photo-crosslinkable component also allowed for improved long-term structural stability of the 3D constructs. Immediately post-UV crosslinking, the constructs will support cells initially. The gradual degradation of the constructs overtime will allow for cellular proliferation and cellular remodeling of the matrix and ECM deposition, without bulk degradation/sudden loss of integrity. Additionally, the slow degradation of the bioink formulations is expected to support slow release of the biochemical cues within the dcECM and ensure diffusion of oxygen and nutrients.

Decellularized tissues contain structural and biochemical signaling cues that are native to the tissue. However, post-processing these are often at lower concentrations compared to the native tissue, therefore there may be strategies needed to enrich the system with specific growth factors. Laponite^®^ is a two-dimensional synthetic magnesium silicate clay. The structure is comprised of disc-shaped particles; the surfaces of which are negatively charged and edges positively charged [57, 58]. Including its role and use as a viscosity modifier, Laponite^®^ also offers significant potential for TE applications and is widely employed as a drug delivery agent [59-61]. The favorable properties and interactions of Laponite^®^ with growth factors offers a promising strategy to use as a delivery vehicle and to overcome the lower ECM concentrations of the dcECM. Bioink formulations were made by loading model proteins FITC-BSA and PDGF-bb. The Laponite^®^ inside the bioink formulation led to favorable drug loading properties as evidenced by the binding affinity of the FITC-BSA to the Laponite^®^, which was visibly present in the system. Release studies of the bioink formulations loaded with FITC-BSA or PDGF-bb showed limited release of the loaded components in a sustained manner overtime. These results indicate the influence of Laponite^®^ discs on the adsorption and potential electrostatic interaction with the loaded biological agents/growth factors [62]. Similar results have also been reported following loading of Laponite^®^-based gels with different growth factors, VEGF and FGF2, and demonstrated their sustained release [63, 64]. The utilization of Laponite^®^ within these bioink formulations provides the ability to supplement dcECM with critical GFs important for regeneration *in vivo.*

Biocompatibility of the developed bioinks were also assessed to determine their suitability for cell culture and TE applications. For OC engineering applications, hBMSCs and chondrocytes were embedded and cultured within the dcECM-based bioink system. Our results also show that the developed bioink encapsulated with primary hBMSCs and chondrocytes exhibited high viability (>94%) after 7 d of culture, thus validating that the UV crosslinking and the additives within our printable bioink formulations can support the survival of stem cells as well as chondrocytes. This algins with previous reports of Laponite^®^ and PEG-DA-based hydrogels that exhibit excellent biocompatibility for various cell types including stem cells and tissue-specific cells, both *in vitro* and *in vivo* [50, 65].

Further, the cartilage regenerative potential of the developed dcECM-based bioink was investigated. The ECM is a complex network of proteins and polysaccharides with tissue-specific composition and architecture that is a critical component of the stem cell niche and for driving the regeneration of damaged tissues and organs. However, many natural and synthetic biomaterial scaffolds are unable to replicate and recapitulate the complexity of the native ECM. The development of decellularization techniques provides the ability for the fabrication of ECM biomaterials that preserve the native structure and composition. In this study, the cartilage regenerative potential of the developed dcECM-based bioink was investigated. It was seen that the developed cartilage tissue-ink resulted in increased levels of proliferation of hBMSCs compared to the tissue-ink formulation with no dcECM (0dcECM/2Laponite^®^/4PEG-DA). This increase in cellular proliferation may be due to the retention of ECM components within the dcECM, allowing for the growth and maintenance of embedded cells. Unlike the hBMSCs, chondrocytes showed a slight decrease in the number of cells, which may be due to the non-proliferative nature of chondrocytes in their natural phenotype or the commitment of terminally differentiated chondrocytes to lay down new ECM [66, 67].

Also, the developed dcECM-based bioink was able to induce MSC chondrogenesis and increase chondrogenic activity of chondrocytes, seen through GAG accumulation and histological staining. When cultured in the dcECM-based bioink (0.5dcECM/2Laponite^®^/4PEG-DA), hBMSCs showed higher level of GAG accumulation compared to the non-ECM bioink formulation (0dcECM/2Laponite^®^/4PEG-DA). It was also noted that the hBMSCs (basal) showed DNA content that plateaued at Day 14, rather than the hBMSCs (chondrogenic) which plateaued at Day 7, due to the earlier transition to differentiated cells which do not proliferate unlike stem cells. Additionally, when cultured in basal medium hBMSCs showed similar sGAG deposition for both groups until Day 14, where ECM bioink demonstrated increased GAG accumulation compared to non-ECM bioink at Day 21. The ECM bioink encapsulated with hBMSCs (chondrogenic), however, showed higher sGAG at an earlier timepoint, Day 14. This may be due to the fact that hBMSCs being progenitor cells, requiring an additional differentiation time to express chondrogenic activity in the absence of chondrogenic-inducing medium. Nevertheless, ECM bioink with hBMSCs cultured in basal medium showed enhanced ECM deposition compared to non-ECM bioink, suggesting the addition of dcECM enhanced hBMSC chondrogenic activity, without the need for chondrogenic additives. For chondrocytes, cartilage tissue-ink encapsulation demonstrated even higher levels of GAG production compared to hBMSCs. The increase in GAG accumulation for chondrocytes was presumably due to the terminally differentiated chondrocytes, which demonstrated greater performance as they do not need to go through the phase of differentiation (unlike hBMSCs). The developed decellularized cartilage-based bioink reveals the ability to promote MSC chondrogenic differentiation and the maintenance of chondrocyte phenotype and chondrogenic activity. This system, with the inclusion of dcECM components, therefore, may have the potential to provide signaling cues for the regeneration of cartilage tissue, as previously reported by other studies where the inclusion of ECM components had the ability to stimulate regeneration.

## 5. Conclusion

We developed a decellularized tissue-based bioink for cartilage and OC engineering. Through the addition of viscosity modifiers, the rheological behavior and printability of the dcECM-based bioink were greatly enhanced, allowing for use as a supportive, printable bioink. A spreadable ink composition was chosen to help form a uniform cartilage layer post-printing. Moreover, inducing a secondary crosslinking mechanism and through interactions imparted by Laponite^®^, the bioink demonstrated stability with prolonged and sustained growth factor release. *In vitro* studies with the chosen bioink, encapsulated with two different cell types, demonstrated the system to support hbMSCs and chondrocyte viability. Moreover, through the addition of the dcECM component, the chondrogenic properties of the bioink were enhanced, accelerating the rate of chondrogenic differentiation and matrix deposition. The improved chondrogenic performance and the tunability of the rheological properties of the bioink suggests the feasibility of designing scaffolds for range of applications, including scaffolds for cartilage tissue repair and regeneration and the fabrication of OC scaffolds by selectively printing/infusing top zone of a porous and mechanically supportive OC template that can support new cartilaginous tissue formation. In conclusion, a Cartilage ink was developed with potential for use in articular cartilage and OC TE.

## Supplementary Material

Supplementary material for this article is available online

## Figures and Tables

**Table 1.** Calculated flow index based on the viscosity data for the cartilage ECM bioinks with and without XG and Laponite^®^ viscosity modifiers.

Sample	Flow index
0.5% dcECM	0.25
0.5% dcECM 0.5% XG	0.27
0.5% dcECM 1% XG	0.17
0.5% dcECM 1.5% XG	0.14
0.5% dcECM 2% Laponite^®^	0.09
0.5% dcECM 3% Laponite^®^	0.1
0.5% dcECM 4% Laponite^®^	−0.04

## Data Availability

All data that support the findings of this study are included within the article (and any supplementary files).
